# An Unusual Case of Ehrlichiosis Manifesting With Hyponatremia, Acute Encephalopathy, and Hemophagocytic Lymphohistiocytosis

**DOI:** 10.7759/cureus.26943

**Published:** 2022-07-17

**Authors:** Taryn Bolling, Alaina S Ritter, Asmita A Gupte

**Affiliations:** 1 Department of Infectious Diseases and Global Medicine, College of Medicine, University of Florida, Gainesville, USA; 2 Division of Infectious Diseases, North Florida/South Georgia Veterans Affairs (VA) Medical Center, Gainesville, USA

**Keywords:** hemophagocytic lymphohistiocytosis, zoonoses, tick-borne diseases, ehrlichia chaffeensis, ehrlichiosis

## Abstract

Ehrlichiosis is a tick-borne infection that has become increasingly more common in the United States in recent years. We present a case of a patient who was found to have confusion, hyponatremia, and hemophagocytic lymphohistiocytosis after contracting *Ehrlichia chaffeensis* following a tick exposure. This unusual presentation emphasizes the need for increased awareness of the varied symptoms of this infection and the importance of obtaining a complete history from patients at risk of vector-borne diseases.

## Introduction

Ehrlichiosis, previously known as human monocytic ehrlichiosis (HME), is a zoonotic disease resulting from an infection with *Ehrlichia chaffeensis*, *E. ewingii*, or *E. muris eauclairensis* [1-2]. While it was initially thought to be a disease of canines, the first human case was discovered in 1986 [3]. It became a reportable disease in 1999, with approximately 200 cases of *E. chaffeensis* noted in 2000 in the US [1]. The number of *Ehrlichia* cases in the US has continued to increase, with 2,093 cases of *E. chaffeensis* reported in 2019 and an overall mortality rate of approximately 1% [1]. *Ehrlichia* infection is typically spread via the bite of an infected tick. *Amblyomma americanum* (lone star tick) is the primary vector of *E. chaffeensis* and *E. ewingii,* while *Ixodes scapularis* (black-legged tick) is the primary vector of *E. muris eauclairensis* [1-2]. *E. chaffeensis*, an obligate intracellular bacterium, is the most common cause of Ehrlichiosis and typically causes mild to moderate disease. Common symptoms can include fever, chills, headache, confusion, and rash, although it can result in more severe disease in immunocompromised patients [1-2]. It is, therefore, important for clinicians to recognize the signs, symptoms, and risk factors for this disease to ensure that patients receive a timely diagnosis and appropriate treatment [1-2]. We present a unique case of a 59-year-old man who contracted *E. chaffeensis* from a tick bite and subsequently developed hyponatremia, encephalopathy, and hemophagocytic lymphohistiocytosis (HLH).

## Case presentation

A 59-year-old man with a past medical history of monoclonal gammopathy of undetermined significance (MGUS), gastroesophageal reflux, and a traumatic brain injury six months prior presented to our hospital emergency department in August with complaints of worsening forgetfulness, dizziness, headache, urinary frequency, and ataxia for three weeks. He also reported a 20-pound weight loss over the past several months. Laboratory data at that time was notable for hyponatremia, thrombocytopenia, and transaminitis. Urinalysis was negative for infection. See Table 1 for a summary of the patient’s labs at the initial presentation compared with his baseline.

**Table 1:** Laboratory results prior to and during admission ^1 ^Hemoglobin and hematocrit could not be processed until day seven of admission due to “interfering substances”

Laboratory test	Results three months prior to presentation	Results at initial presentation	Results on Day 1	Results on Day 7	Results at discharge (Day 9)	Reference Range
White-cell count	4.71 k/mm^3^	4.64 k/mm^3^	3.41 k/mm^3^	6.65 k/mm^3^	7.35 k/mm^3^	4.6-10.8 k/mm^3^
Hemoglobin	12.1 g/dL	- ^1^	- ^1^	10.5 g/dL	10.0 g/dL	13.9-18 g/dL
Hematocrit	33.7%	- ^1^	- ^1^	28.2%	27.4%	41-52%
Platelet count	213 k/mm^3^	47 k/mm^3^	54 k/mm^3^	194 k/mm^3^	387 k/mm^3^	130-440 k/mm^3^
Blasts	0 k/mm^3^	0.05 k/mm^3^	0 k/mm^3^	0 k/mm^3^	0 k/mm^3^	0-0 k/mm^3^
Creatinine	0.9 mg/dL	1.3 mg/dL	1.3 mg/dL	0.9 mg/dL	0.8 mg/dL	0.5-1.2 mg/dL
Sodium	142 mmol/L	122 mmol/L	120 mmol/L	136 mmol/L	137 mmol/L	135-145 mmol/L
Potassium	4.3 mmol/L	3.9 mmol/L	3.8 mmol/L	3.8 mmol/L	4.1 mmol/L	3.5-5.0 mmol/L
Chloride	106 mmol/L	86 mmol/L	84 mmol/L	98 mmol/L	99 mmol/L	98-108 mmol/L
Calcium	9.3 mg/dL	8.0 mg/dL	7.9 mg/dL	8.9 mg/dL	8.8 mg/dL	8.4-10.5 mg/dL
Total bilirubin	0.5 mg/dL	0.6 mg/dL	0.3 mg/dL	0.6 mg/dL	0.5 mg/dL	0.0-1.3 mg/dL
Alkaline phosphatase	46 U/L	39 U/L	42 U/L	59 U/L	65 U/L	0-125 U/L
Aspartate aminotransferase (AST)	33 U/L	280 U/L	305 U/L	277 U/L	150 U/L	0-45 U/L
Alanine aminotransferase (ALT)	16 U/L	61 U/L	77 U/L	196 U/L	145 U/L	0-40 U/L
Fibrinogen	-	230 mg/dL	-	-	-	207-451 mg/dL
D-dimer	-	2.75 ug/mL	-	-	-	<=0.49 ug/mL
INR	-	1.1	1.1	-	-	0.9-1.2
Prothrombin time	-	13.9 sec	13.8 sec	-	-	12.2-14.8 sec
Triglycerides	-	238 mg/dL	-	-	142 mg/dL	0-149 mg/dL
Ferritin	-	61,781 ng/mL	-	7,625 ng/mL	-	30-285 ng/mL

The patient left against medical advice before additional workup could be performed, but he returned to the emergency department for admission the next day due to persistent symptoms. Upon repeat presentation (day one of admission), vital signs were notable for a temperature of 100.2°F. He was otherwise hemodynamically stable and denied any fevers, chills, numbness or tingling in the extremities, nausea, vomiting, abdominal pain, hallucinations, or dysuria. Physical and neurologic exams were significant only for confusion and an antalgic gait. Additional laboratory findings at that time (Table 1) were notable for a ferritin of 61,781 ng/mL (reference range 30-285 ng/mL) and triglycerides of 238 mg/dL increased from 59 mg/dL one year prior (reference range 0-149 mg/dL). CT head was notable for a focus on age-indeterminate hypodensity in the right cerebellar hemisphere concerning a possible ischemic event. This finding was new within the past year. Subsequent MRI head showed a multifocal contusion consistent with his known traumatic brain injury and a poorly visualized right cerebellar hypodensity concerning ischemia. He was managed supportively for a possible recent stroke. 

**Figure 1:**
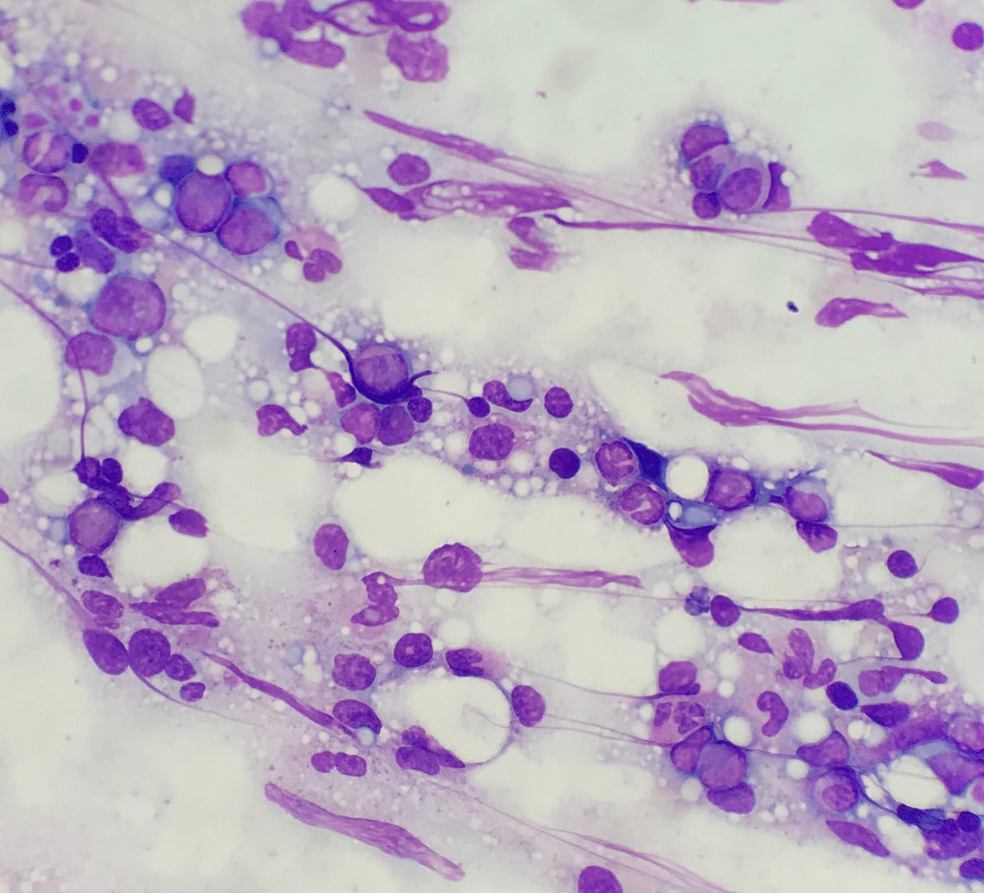
Bone marrow aspirate Wright-Giemsa stained bone marrow aspirate slides showed scattered histiocytic cells with vacuolated cytoplasm demonstrating phagocytosis of cellular elements, including platelets, erythroid cells, and cell fragments (600x)

On day two of his admission, the patient developed fevers to 101-102°F and was started empirically on intravenous vancomycin and cefepime. Upon further questioning, the patient revealed that he had removed two engorged ticks from his right axilla and upper back one month before presentation while fishing locally in Florida. Empiric doxycycline 100 mg twice daily was started for possible tick-borne disease. Hematology was consulted for a work-up of his thrombocytopenia and elevated ferritin. Bone marrow biopsy was performed on day three of admission and revealed increased macrophages containing phagocytic debris consistent with hemophagocytic lymphohistiocytosis. The bone marrow biopsy was also notable for a monoclonal kappa, and plasma cell infiltrate accounting for 15%-20% of marrow cellularity, favoring a low-grade, indolent myeloma consistent with his known history of monoclonal gammopathy of undetermined significance (MGUS) (Figure 1).

Serologic testing ultimately yielded a positive *Ehrlichia chaffeensis* IgG (titer 1:128), negative IgM (<1:20), and positive *Ehrlichia chaffeensis* DNA polymerase chain reaction (PCR), thus confirming the diagnosis of ehrlichiosis. A Lyme enzyme-linked immunosorbent assay (EIA) screen, HIV ag/ab, syphilis IgG/IgM and RPR, hepatitis C antibody, hepatitis B surface antigen/surface antibody/core antibody, rocky mountain spotted fever IgG and IgM, and babesiosis IgG and IgM were all negative. Epstein-Barr virus (EBV) serology was consistent with past exposure. 

His fevers and mental status improved, and he was discharged on day nine of his admission to complete 14 total days of oral doxycycline. See Table 1 for laboratory findings at the end of his hospital stay. He did not receive any additional therapies for HLH during his hospitalization or after discharge. Upon follow-up after completion of his antibiotic course, the patient reported full resolution of his symptoms. All other laboratory findings had returned to baseline.

## Discussion

Ehrlichiosis is primarily found in the southeastern and south-central United States. This coincides with the geographic distribution of *Amblyomma americanum*, the lone star tick, which carries *E. chaffeensis* and *E. ewingii*. *E. muris eauclairensis* is a less common cause of ehrlichiosis, with cases first reported in 2009. *E. chaffeensis*, the organism identified in this patient, is involved in the majority of Ehrlichiosis cases. The increasing trend in cases from 2000 to 2019 in the US is likely due to a combination of warming climates and increased recognition and diagnosis of this infection. The majority of cases occur during the summer months, typically June and July, given that nymphal and adult lone star ticks are the most abundant during this time. Risk factors for ehrlichiosis include male gender, increased age (primarily ages 60-69), and immunocompromise [1-2].

When an infected tick bites a human host, the elementary body of the organism undergoes endocytosis in the host cell. Once inside, it replicates and matures into the reticulate body or reticulate core. It subsequently differentiates into a morula before differentiating back into an elementary body/dense core, which spreads infection throughout the host [2]. *E. chaffeensis* typically infects human monocytic cells, including monocytes and macrophages. Symptoms vary in severity and typically stem from the inflammatory response of the host to the infection. Patients frequently present with fever, chills, headache, myalgias, nausea, vomiting, malaise, and confusion. Some patients may also present with a rash, although this is more common in children. Classically, the rash presents as maculopapular with some petechiae and/or diffuse erythema. In patients with immunocompromise, symptoms may be more severe and can include acute respiratory distress syndrome, meningoencephalitis, septic shock, disseminated intravascular coagulation, acute renal failure, gastrointestinal bleeding, and myocarditis [4]. The onset of symptoms typically begins one to two weeks after the initial tick bite. Obtaining a detailed history and physical exam is important, as many of these symptoms are non-specific [2]. Travel history, exposures, and risk factors for tick-borne illness should also be assessed [1].

Laboratory findings are often notable for leukopenia, thrombocytopenia, hyponatremia, and transaminitis [2]. Diagnosis may be confirmed with PCR, which is most sensitive in the first week of the disease, with decreasing sensitivity once antibiotics are initiated. Microscopy may show morulae in the cytoplasm of leukocytes. Indirect immunofluorescence antibody assays for immunoglobulin G may also be performed on two convalescent serum samples collected two to four weeks apart. It is important to note that antibody titers are sometimes negative in the first week of illness and can remain positive for several weeks after the disease has been treated. Cross-reactivity may occur with *Anaplasma phagocytophilum* as a result of similar antigens [1].

Hemophagocytic lymphohistiocytosis is a rare complication of Ehrlichiosis. HLH is a systemic inflammatory illness that results from a dysregulated immunologic response. Pathogenesis primarily involves CD8 cytotoxic T-cells and natural killer (NK) cells, which are activated and proliferate uncontrollably. This stimulates the proliferation of macrophages and a subsequent release of numerous cytokines leading to a cytokine storm. This cytokine storm results in phagocytosis of bone marrow hematopoietic cells in various organs. Depending on the patient, this may manifest as fever, lymphadenopathy, hepatosplenomegaly, pancytopenia, and hyperferritinemia. HLH may be fatal in some cases, and so it is important to recognize these clinical signs in order to initiate appropriate treatment. HLH can be a primary disease resulting from underlying genetic defects (familial HLH), or it can be secondary due to underlying infection or malignancy [5]. Infection with EBV is likely the most frequent viral cause of HLH, although HIV infection is another common cause. Bacterial pathogens account for less than 10% of cases, with *Mycobacterium tuberculosis* being the most common. Ehrlichiosis represents a rare cause of HLH, and the true mechanisms of this association remain unclear. It has been postulated that since *Ehrlichia spp*. are gram-negative bacteria without a lipopolysaccharide wall, NK cells are unable to recognize them to mount an appropriate response. This may allow for infection with subsequent development of HLH [6].

The standard recommended treatment course for Ehrlichiosis is oral doxycycline 100 mg twice daily for seven to 14 days. In cases of doxycycline allergy, there is some limited clinical data to support the use of oral rifampin, which has in vitro bactericidal activity against *Ehrlichia spp*. A case report from 2016 describes a 64-year-old woman with a known allergy to doxycycline who developed transaminitis, leukopenia, and thrombocytopenia after a tick bite. Her *E. chaffeensis* PCR returned positive, and she was successfully treated with seven days of oral rifampin 300 mg twice daily with complete resolution of her symptoms [7]. Chloramphenicol has also been considered as an alternative therapeutic option, although antibiotic susceptibilities indicate this drug may be ineffective against other species of *Ehrlichia*, including *E. chaffeensis* [7]. In addition to receiving antibiotic treatment, patients should also be advised to engage in preventative measures to avoid reinfection, which include avoiding high-risk outdoor areas, using insect repellents, wearing clothing that covers exposed skin and is light-colored to improve visualization of ticks, and performing self-checks for ticks [2]. 

Timely diagnosis and treatment of ehrlichiosis are essential to ensure optimal patient outcomes. Ehrlichiosis can be fatal if left untreated and early initiation of therapy is key to preventing clinical deterioration. A study by Hamburg et al. looked at hospitalized patients who had early initiation of doxycycline (within 24 hours of admission) compared to those who did not. Out of 46 total patients with a positive serum PCR for *Ehrlichia spp*., 18 were started on doxycycline within 24 hours of admission, and 28 were started greater than 24 hours after admission. Patients with delayed initiation of therapy were found to have significantly higher rates of complications, including admission to the intensive care unit and the need for mechanical ventilation and vasopressors. This group of patients also had longer hospital stays compared with the patients who received early therapy (20.9 ± 14.1 days vs. 8.9 ± 2.7 days; p = 0.001) [8]. These results further support having a high index of suspicion for ehrlichiosis and other tick-related diseases in endemic areas. Clinicians should also have a low threshold to initiate doxycycline empirically while awaiting confirmatory laboratory data. Our patient was started on oral doxycycline on his second day of hospitalization, was discharged on the ninth day of his hospitalization, and ultimately made a full recovery. 

## Conclusions

We report an unusual case of ehrlichiosis after a tick bite presenting with hyponatremia, encephalopathy, and HLH diagnosed on bone marrow biopsy. Oral doxycycline was started empirically while awaiting confirmatory serologic testing, which was ultimately positive for *E. chaffeensis*. The patient’s symptoms completely resolved after completing 14 days of doxycycline therapy. This case report highlights important clinical features of this infection that may assist clinicians in making a timely diagnosis and starting appropriate treatment.
